# Hemicellulose-Based Film: Potential Green Films for Food Packaging

**DOI:** 10.3390/polym12081775

**Published:** 2020-08-07

**Authors:** Yuelong Zhao, Hui Sun, Biao Yang, Yunxuan Weng

**Affiliations:** 1 College of Chemistry and Materials Engineering, Beijing Technology and Business University, Beijing 100048, China; 13552134315@163.com (Y.Z.); ybiao@th.btbu.edu.cn (B.Y.); wyxuan@th.btbu.edu.cn (Y.W.); 2 Beijing Key Laboratory of Quality Evaluation Technology for Hygiene and Safety of Plastics, Beijing Technology and Business University, Beijing 100048, China

**Keywords:** hemicelluloses, film, food packaging, modification

## Abstract

Globally increasing environmental awareness and the possibility of increasing price and dwindling supply of traditional petroleum-based plastics have led to a breadth of research currently addressing environmentally friendly bioplastics as an alternative solution. In this context, hemicellulose, as the second richest polysaccharide, has attracted extensive attention due to its combination of such advantages as abundance, biodegradability, and renewability. Herein, in this review, the latest research progress in development of hemicellulose film with regard to application in the field of food packaging is presented with particular emphasis on various physical and chemical modification approaches aimed at performance improvement, primarily for enhancement of mechanical, barrier properties, and hydrophobicity that are essential to food packing materials. The development highlights of hemicellulose film substrate are outlined and research prospects in the field are described.

## 1. Introduction

Plastics are the most demanded packaging material for the food industry due to the unique combination of their properties like light weight, low cost, convenient processability and excellent chemical resistance. The market for food packaging materials is expected to reach $380 billion by 2022 due to the increased demand for convenience foods [1]. Among them, plastic consumption is the largest, amounting to more than 30%. Currently, 99% of plastic bags are made of petroleum-based materials [2], which can persist for hundreds of years in nature. So far, more than 6 billion metric tons of plastic waste has accumulated around the world. The use of petroleum-based plastics not only consumes non-renewable resources, but also causes serious pollution to the environment. In light of this, the food packaging community is starting to search for bio-alternatives to solve this problem.

Bioplastics, which include bio-based plastics and biodegradable plastics, accounted for less than 1% in 2017, and are expected to reach 40% by 2030. Bioplastics used as packaging accounted for more than 60% [3]. In the field of food packaging film, lignocellulose from renewable resources has gained attention because of its potential to replace traditional film materials. Its use can reduce the consumption of petroleum-based materials, and at the same time its biodegradability helps to reduce environmental pollution. 

Bio-based food packaging films predominately originating from polysaccharides are currently being extensively explored [4,5]. Lignocellulose is rich in polysaccharides. Lignocellulosic materials are the main components of biomass, accounting for about half of the matter produced by photosynthesis [6]. Lignocellulose is mainly composed of cellulose, hemicellulose, and lignin. Application of cellulose in the field of food packaging has been studied. For example, cellulose nanocrystals has already found its preliminary application in food packaging due to its renewability, unique nanoscale structure, biocompatibility, and easy surface modifications [7,8]. Hemicellulose with content being second only to cellulose has shown a broad application prospect due to its wide sources, renewability and biodegradability. Unlike cellulose having homogeneous glycan structure composed entirely of β-(1→4)-glucan connecting to the dextran chain, hemicellulose is composed of a variety of glycan structures, with degree of polymerization ranging from 100 to 300. 

**Figure 1:**
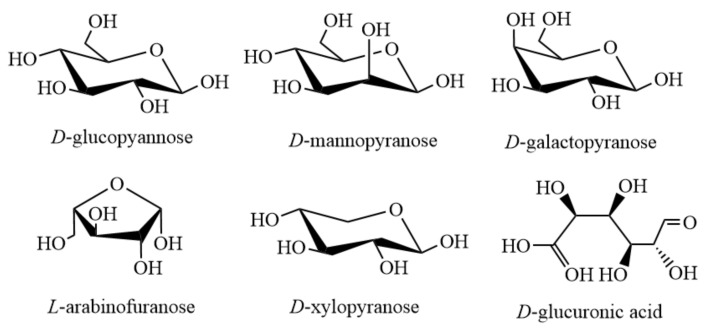
Main structural units of hemicellulose.

Main sugars contained in hemicellulose include xyloglucan, xylan, mannannan and glucomannan, β-(1→3,1→4)-glucan, etc., among which, other sugars except β-(1→3,1→4)-glucan, exist in the cell walls of all terrestrial plants. The detailed structure and abundance of hemicellulose vary greatly among different species and cell types [9]. The basic glycoelements of the polysaccharides with different structures mentioned above are *D*-glucopyannose, *D*-mannopyranose, *D*-galactopyranose, *L*-arabinofuranose, *D*-xylopyranose and *D*-glucuronic acid [10] (Figure 1).

At present, the methods of extracting hemicellulose from plants can be divided into physical pretreatment and chemical pretreatment. The former includes steam explosion [11], hot water extraction [12], ultrasound, and microwave-assisted methods [13]. The latter involves the use of alkali [14,15], acid [16], and organic solvent [17]. Jin and co-workers [18] analyzed the yield of hemicellulose under different conditions by using alkali or the alkaline peroxide solution method and the two-step alkali extraction-delignification method (Table 1). They found that hemicellulose obtained by different methods had different structures, giving them different properties. The continuous improvement of extraction methods has made it possible for hemicellulose to gain practical application. Exploration of hemicellulose films can be traced back to 1949, when Smart and Whistler [19] made film materials from acetyl hemicellulose. Because of its defects such as poor mechanical properties and barrier properties, the modification of hemicellulose film has long been a research thrust.

## 2. Hemicellulose Film Matrix

**Figure 2:**
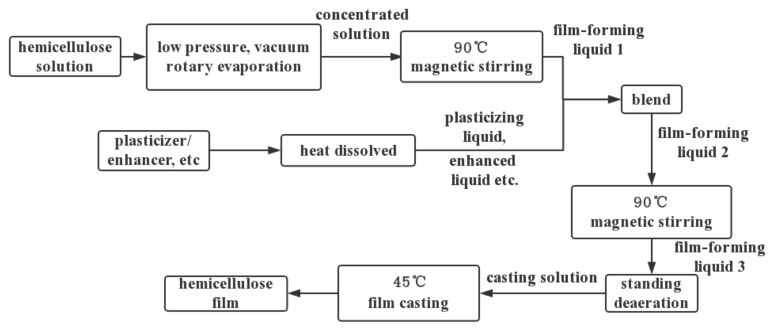
Flow chart of hemicellulose film preparation.

Traditional plastic food packaging materials including edible film are mostly non-renewable petroleum-based materials. Hemicellulose originating from renewable biomass resources share the advantages of being biodegradable in the environment, biocompatible, and can be therefore used as both food packaging and edible film. Compared with cellulose, hemicellulose has a relatively low molecular weight, and thus a low strength. However, hemicellulose has unique advantages due to its functional hydroxyl group and various structures, which are easy to modify to meet different requirements. Commonly used biomass film substrates include flat film and hollow film. Hemicellulose is usually used in the form of a flat film, generally prepared by blending flow casting [20], casting [21] and drying film-forming solution on a mold [22]. The commonly used preparation process is shown in Figure 2.

Examples of property requirements for food packaging materials are acceptable mechanical properties, good barrier, and flexibility. Unmodified hemicellulose films often show poor mechanical properties due to their branched and amorphous structures. Mechanical properties of hemicellulose films based on different biomass sources vary considerably [23], as shown in Table 2.

In addition, with regard to its use as a packaging material, hemicellulose films also exhibit poor compatibility with traditional plastics and poor thermal stability [24].

Moreover, a large number of hydrophilic hydroxyl groups on the side chain make hemicellulose film susceptible to absorption of moisture, resulting in poor performance when used in humid environment [25]. Poor mechanical strength and barrier properties make unmodified hemicellulose film unsuitable for use as food packaging material. To this end, physical and chemical modification of hemicellulose have been extensively explored.

## 3. Modification of Hemicellulose

Efforts have been made to improve the performance of hemicellulose film intended for use as packaging material. At present, the most common methods are physical and chemical modification.

### 3.1. Physical Modification

In a typical physical modification, small molecular and/or high molecular substances is evenly distribute into hemicellulose matrix by blending, with the aim to change the microstructure and composition of hemicellulose film through the action of non-chemical bonds, and thus to improve its mechanical properties.

#### 3.1.1. Plasticizer

Addition of plasticizers have been approved effective in improving film-forming property and mechanical properties of hemicellulose film to a certain extent. Commonly used plasticizers include glycerin, xylitol, and sorbitol [10,32]. Plasticizers help to achieve better film-forming ability and film flexibility by breaking hydrogen bonds between polymer chains, thus increasing free volume and chain fluidity.

In selecting appropriate plasticizer for the hemicellulose film, Xu and co-workers [33] reported the comparison of mechanical properties of cellulose nanofibers (CNF)-hemicellulose/chitosan composite films plasticized by different types of plasticizers, i.e., glycerin, xylitol, and sorbitol. It was found that with the increase of plasticizer concentration, the elongation at break of the composite increases obviously, while the tensile strength decreases. Glycerin showed the best plasticization effect, probably because the relatively small size facilitates its insertion and fixation in the hemicellulose/chitosan matrix; Addition of sorbitol gave rise to the highest tensile strength. However, the addition of plasticizer enhanced the hydrophilicity of the film, leading to slightly increased moisture permeability of the film. 

Besides the improvement in elongation at break, addition of plasticizer was also reported to improve the barrier properties. When Hartman and co-workers [34] studied the plasticization of sorbitol on *O*-acetylgalactomannan, they found that the formation of rigid polymer chain network lead to lowered free volume between the chains, thus improving the barrier performance under wet conditions. A reduced oxygen permeability of 2.0 cm^3^·μm·m^−2^·d^−1^·kPa^−1^ can be achieved, which is of the same level as traditional petroleum-based materials such as polyethylene vinyl alcohol and polyvinylidene chloride.

Despite the improved comprehensive performance compared with unmodified hemicellulose film, plasticized hemicellulose film is still inferior to traditional petroleum-based materials and it is thus difficult to meet the needs of food packaging materials.

#### 3.1.2. Reinforcing Agent

To further improve mechanical properties, reinforcements is usually blended with hemicellulose film. Reinforcing agents may change the group distribution and structure by hydrogen bonding with the hydroxyl group in hemicellulose, yielding polymer systems with enhanced mechanical properties.

Guan and co-workers [35] added montmorillonite (MMT), a commonly used reinforcing agent into quaternized hemicellulose (QH) to prepare nanocomposite films and the tensile strength and elongation at break can reach 19.8MPa and 0.5% [36], respectively. To further improve the mechanical properties of QH/MMT composite film, Chen and co-workers [36] added polyvinyl alcohol (PVA) and chitin nanowhisker (NCH) to the QH/MMT film (Table 3). The results showed that the composite films exhibited a substantial rough surface because the addition of PVA and NCH. Due to hydrogen bonding and electrostatic interaction, the tensile strength, optical transparency and oxygen barrier properties of the composites were significantly improved. Chitosan (CS) was also used to enhance the QH/MMT composite film [37]. Compared with the large amount of PVA and NCH loading, the mechanical properties of QH/MMT composite films can be greatly enhanced with only a small amount of CS addition (Table 3). At a QH:MMT:CS mass ratio of 1:1:0.08, the tensile strength of composite film can reach 57.8 MPa. At the same time, the composite shows higher optical transparency, great thermal performance, lower oxygen permeability, and water vapor permeability. Since PVA, NCH, and CS are either water soluble or biopolymers, their use as enhancement components with good biocompatibility are beneficial to the preparation of environmentally friendly composite film.

Cellulose nanofiber (CNF) is a green, multi-functional nanomaterial with great attraction due to its unique mechanical properties and high aspect ratio. Typical characteristics of nanoscale cellulose include high strength, high stiffness, low weight, degradability, regeneration and so on. Fibres are known reinforcing agents and the addition of fiber polymers can obviously improve the mechanical properties of hemicellulose films. Agnes and co-workers [38] mixed acetylated xylan (AcAx) with cellulose nanofibers for subsequent film casting. They found that increasing CNF loading gradually decreases the tensile strain at fracture but increases film stiffness and fracture stress (Table 4). At 10% CNF loading, the film showed the highest comprehensive mechanical performance, with the ultimate strength and young’s modulus increased to 93 MPa and 3360 MPa, respectively, compared with pure AcAx film (65MPa and 2190MPa). In addition, CNF-modified AcAx showed a moisture absorption rate of less than 8% under 97% relative humidity at room temperature. For hemicellulosic/chitosan system, the addition of 5% CNF could increase the tensile strength of the film by 2.3 times [33]. 

The above-mentioned reinforcers effectively enhanced the strength but usually inevitably reduced the toughness of the film at the same time. It was found that cellulose nanocrystalline (CNC) is capable of overcoming this disadvantage. Pereira and co-workers [39] added CNC to wheat straw hemicellulose for film preparation, and found that the addition of CNC effectively improved the tensile strength, tensile modulus, and moisture resistance of the blend film (Table 5). A possible explanation is that with the addition of CNC, a large number of hydrogen bond networks are generated in the composite film [40]. Huang and co-workers [41] compared cationized CNC with non-cationized CNC, and found that the number of hydroxyl groups decreased after cationization, which weakened the hydrogen bond effect. Therefore, cationized CNC has a less effect on improving the mechanical properties of film compared with CNC. However, the increase of carboxymethyl group improves its dispersion in the matrix, resulting in a smoother film surface and better thermal stability.

In addition to the several strengthening agents mentioned above, examples of commonly used strengthening agents include nano silica, corn starch [42], and sodium carboxymethyl cellulose (CMC) [43]. The physical modification of hemicellulose film via blending is effective in elevating mechanical properties to a level needed for use as packaging material. However, some hydrophilic groups will also be introduced, which cannot get around the drawbacks associated with hydrophilicity that is inherent to hemicellulose. This modified hemicellulose is difficult to be directly used in the field of food packaging.

### 3.2. Chemical Modification

A large number of active groups, such as hydroxyl, carboxyl, and carbonyl, on the main and side chains of hemicellulose can be used as reaction sites for modification through a series of chemical reactions. These modifications provide a way to improve the properties of hemicellulose, especially barrier properties, thus making it more suitable for food packaging.

#### 3.2.1. Esterification

**Figure 3:**
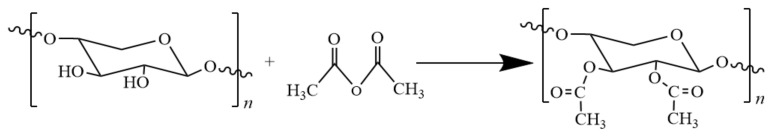
Acetylation of hemicellulose.

Esterification is the reaction of alcohol with carboxylic acid or one of its derivatives to form ester. The derivative may be an acid chloride or an anhydride. Esterification can reduce the tendency of hydrogen bond network formation by replacing hydroxyl with hydrophobic ester to increase the flexibility and hydrophobicity of hemicellulose films [44]. Esterified hemicellulose is usually synthesized with acid anhydride or acyl chloride as the esterification reagent, and acetylated hemicellulose is commonly synthesized by using acetic anhydride (Figure 3). Acetylated hemicellulose has been widely used because of its excellent hydrophobicity.

However, this esterification reaction produces HCl or carboxylic acid as by-products, which leads to serious acid degradation of hemicellulose, thus affecting its use [45]. To prevent the formation of acids, Peng and co-workers [46] obtained a flexible hemicellulose film by using cycloanhydride for esterification reaction. However, the presence of a large number of carboxyl groups makes the film hydrophilic, and thus unsuitable for food packaging.

Hirose and co-workers [47] proposed a transesterification reaction based on vinyl ester when studying the esterification reaction of cellulose. Compared with traditional esterification of anhydride or acyl chloride, ethylene ester-based esterification is a mild acid-free reaction. Following similar train of thought, Zhang and co-workers [48] obtained laurate hemicellulose (LHs) via transesterificaion of hemicellulose with vinyl laurate, using ionic liquid 1-ethyl-3-methylimidazolium acetate as the reaction medium. The introduction of hydrophobic chain greatly improved the solubility of LHs. LHs films with good mechanical properties and hydrophobicity were prepared by direct solvent casting. Solvent evaporation leaves LHs film with surface of orderly and dense honeycomb structure; and the grafted long chain of lauric acid promotes the formation of the dense network of cross sections, thus rendering the LHs film good oxygen and water barrier capability. The water vapor permeability and oxygen permeability were found to be as low as 1.59 ± 0.07 (10^−10^·g/m·s·Pa) and 1.21 ± 0.04 (cm^3^·μm·m^−2^·d^−1^·kPa^−1^), respectively. The laurate grafted chain also enhanced the antioxidant activity of laurate, and played a role in the preservation of food in the package.

**Figure 4:**
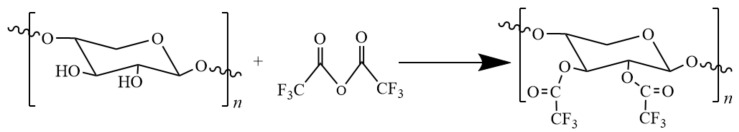
The fluorination of hemicellulose.

Fluorination is also a commonly used hydrophobicization method. Fluorination modification provides polymer substrates with unique surface properties. Fluorinated films show excellent chemical resistance and thermal stability in addition to superhydrophobicity. Grondahl and co-workers [49] performed fluorination of arabinoglycan with gaseous trifluoroacetic anhydride (Figure 4).

The contact angle of the fluorinated arabian xylan surface was determined to be about 70°, much higher than that of un-fluorinated arabian xylan. Though material surface is generally defined as hydrophobic when the contact angle is greater than 90°, the average water content after fluorination decreases from 18% to 12%, indicating a certain decrease in hydrophilicity. One limitation with hemicellulose fluorination is the partial hydrolysis of trifluoroacetate groups after contact with water, which may limit the use of trifluoroacetate groups grafted on hemicellulose.

Surface fluorination obviously improved the hydrophobicity of materials and it is of great significance to find a new fluorination method for hydrophobic modification of hemicellulose. Fluorinated surface can also be obtained by exposing hemicellulose film to a fluorinated plasma gas (e.g., CF_4_). Compared to conventional chemical reaction fluorination, plasma fluorination of polymers was proved to be more direct [50]. The application of this method in modification of hemicellulose film calls for further study.

#### 3.2.2. Etherification

Etherification is the reaction between alcohol and alkylating agent in the presence of alkali. Commonly used alkylating agents include alkylsulfonates, epoxides, and alkyl halides (chlorides, bromides and iodides). Compared with ester bonds which readily hydrolyze, especially under alkaline conditions, ether bonds are more stable in chemical properties. However, the high pH environment required for etherification will result in partial degradation of hemicellulose. To avoid the degradation, etherification of hemicellulose is usually carried out in a mixture of water and organic solvents (ethanol or toluene) [51].

Since epoxides have an unstable oxy-containing ternary ring, adjacent carbons are prone to losing electrons, which makes epoxides relatively reactive to hemicellulose. Nypelö and co-workers [52] achieved the functionalization of galactoglucomannan (GGM) with butyl glycidyl ether as the alkylation reagent. The thus-functionalized GGM showed significantly decreased water absorption, indicating an increased hydrophobicity. Meanwhile, through dynamic mechanical analysis, Härdelin and co-workers [53] found that the functionalization of GGM improved both thermal performance and mechanical properties of the modified film. The modified film showed a reduced glass transition temperature (*T*g), which makes the film easier to process. Compared with that of unmodified GGM, elongation at break of the modified composite film increased from 2% to 12%. In addition to much improved hydrophobicity, the etherification reaction resulted in a more ductile material than the unmodified GGM.

**Figure 5:**
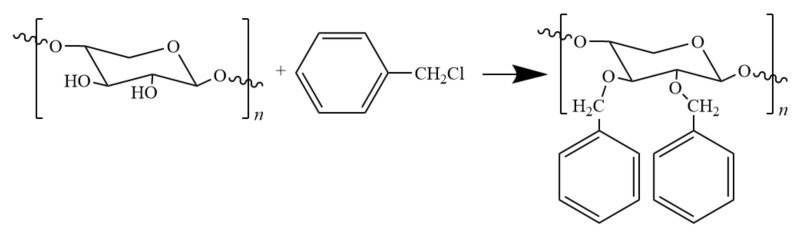
Benzyl reaction of hemicellulose with benzyl chloride.

In the etherification modification of hemicellulose, benzylation was found to afford modified hemicellulose with excellent thermal and mechanical properties as well as good hydrophobicity. Hartman and co-workers [54] obtained BnAcGGM by reacting *O*-acetyl galactoglumanannan (AcGGM) with benzyl chloride (Figure 5). 

The BnAcGGM film showed excellent toughness and transparency, but was less resistant to oxygen than AcGGM film. Ren and co-workers [55] prepared hemicellulose film by reaction with benzyl chloride in ethanol/water system in the presence of sodium hydroxide. The introduction of benzyl not only improved the hydrophobicity of the film, but also brought the decomposition temperature of the film to the maximum, i.e., 298 °C.

#### 3.2.3. Grafting Modification

**Figure 6:**
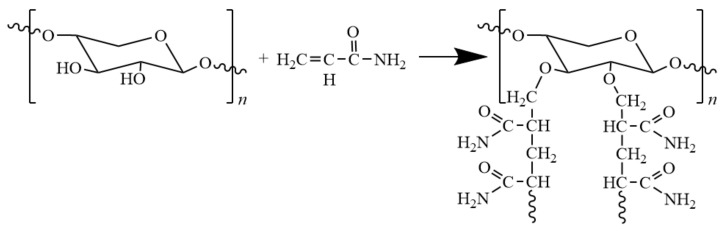
Scheme of the preparation of hemicelluloses-g-polyacrylamide.

Grafting polymerization offers an approach to introducing functional groups into hemicellulose for the purpose of improving properties needed for certain applications. Grafting polymerization serves to reduce the formation of hydrogen bond network, which leads to their hydrophobicity and increased solubility in organic solvents. Additionally, the change of structure also affects the thermal and mechanical properties of hemicellulose-based materials. Common monomers are acrylonitrile, methacrylic acid, and acrylamide (Figure 6). For example, Du and co-workers [56] grafted polyacrylamide to hemicellulose with as cross-linking agent *N*,*N*-methyl-diacrylamide in potassium persulfate/*N*,*N*,*N*′,*N*′-tetramethylenediamine (KPS/ TMEDA) redox system. The grafted films have an elongation at break as high as 61%, and oxygen permeability of 8.75 cm^3^·μm·m^−2^·d^−1^·kPa^−1^, showing excellent stretchability and oxygen barrier property.

Inspired by this approach, Farhat and co-workers [57] designed hemicellulose thermoplastic by ring-opening grafting of polycaprolactone (PCL) onto hemicellulose. PCL grafts act as an internal plasticizer to convert hemicellulose into thermoplastic material with good processability. The PCL-grafted hemicellulsoe has a water contact angle of up to 81°, showing a certain degree of hydrophobicity. In addition, the modified hemicellulose is also biodegradable and can be used as raw material for further processing design for the bioplastic and other industries.

In a series of studies on grafting hemicelluloses, Börjesson and co-workers [58] provided a new route. They innovatively grafted salt-containing azacyclic butadiene groups onto hemicellulose films. Because azacyclobutadiene salts may contain various groups, the graft modification of hemicellulose may be further extended by this method to offer various possibilities. This approach to introduce functional groups into hemicellulose simply by graft modification indicates a new direction for the development of hemicellulose film materials.

#### 3.2.4. Cross-Linking

Modification via crosslinking provides polymers with resistance to thermal degradation and increased resistance to cracking in liquids and other harsh environments. Crosslinking reduces solubility of hemicellulose in liquid and improves water resistance [59]. Hemicellulose can be cross-linked by chemical crosslinkers or irradiation [60,61,62].

**Figure 7:**
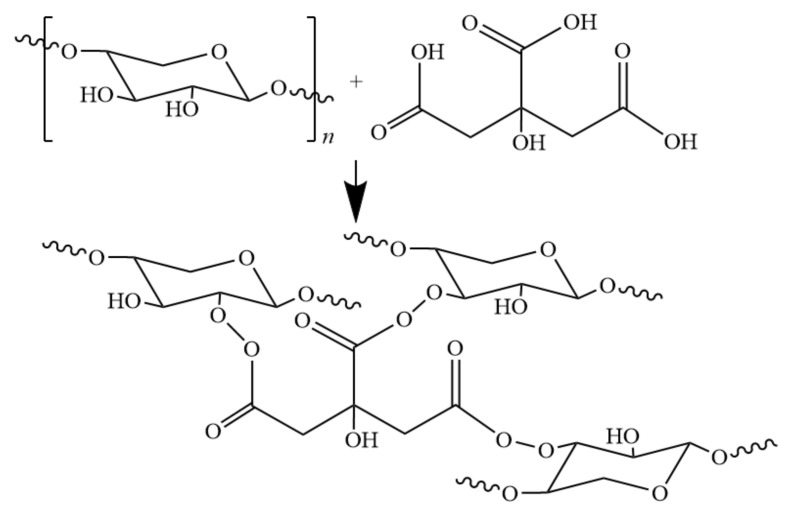
Possible cross-linking reaction between CA and hemicellulose.

When using citric acid as cross-linking agent to treat hemicellulose film, since citric acid is both esterifying agent and cross-linking agent, a special esterified cross-linking structure is produced, which improves the performance of modified hemicellulose film [63,64]. Shao and co-workers [65] presented possible reaction equations in their study of citric acid crosslinking (Figure 7) and studied the barrier properties of the film. The esterified cross-linking structure increases the contact angle of the film material to 87.5°, with oxygen permeability decreased from 1053 cm^3^·μm·m^−2^·d^−1^·kPa^−1^ to 1.8 cm^3^·μm·m^−2^·d^−1^·kPa^−1^. The modified hemicellulose film showed excellent thermal stability, hydrophobicity, and oxygen barrier properties; Mechanical properties was also improved to some extent compared with the unmodified hemicellulose film. Modification via esterification/crosslinking mechanism provides a new research direction for modification of hemicellulose.

Inspired by the tile structure of the natural pearl layer, Huang and co-workers [66] first blended galactomannan (GM) and grapheme oxide (GO) to obtain GM/GO composite film, and further prepared the GM-based hybrid with shell-like structure by in-situ reduction cross-linking with borates. This brick-concrete structure and borate crosslinking enhanced the tensile strength of the composite film with 3% GO loading by 2.4 times compared with the unmodified GM film. The composite film showed good mechanical properties, and certain barrier property. This biomimetic structure provides a new idea for the design of hemicelluloe-based composite materials.

## 4. Application in Food Packaging

As mentioned above, the food industry is the largest consumer of packaging materials. Films used in food packaging aim to protect food from the impact of external environment, such as pressure, vibration, oxygen, water vapor, and microbial effects. The mechanical properties and barrier properties of packaging film are of major importance for packaged foods.

Hemicellulose film is considered a potential alternative to petroleum-based film. Although it is environmentally friendly and can solve the pollution problems caused by plastics to some extent, it has not been further applied due to its poor mechanical properties and barrier properties. After physical and chemical modification, the mechanical properties and barrier properties of hemicellulose films are obviously improved. Modified hemicellulose film has barrier properties comparable to traditional petroleum based film, but much lower mechanical properties, especially the elongation at break (Table 6).

Modified hemicellulose film as packaging material is essentially in the research stage and has also found preliminarily application. When preserving green peppers by using laurate hemicellulose film, compared with traditional cling film, the color of green chilies remained unchanged and the weight dropped at the same level. This is due to the film of good oxygen barrier and the moderate water vapor permeability [48].

Food preservation is usually a long-term process, so the long-term stability of packaging is also important. When Susanna and co-workers [73] studied the plasticized film made from oat spelt arabinoxylan and spruce galactoglucomannan, they found that although the mechanical properties of the film slightly deteriorated after four months of use, water vapor permeability decreased significantly and oxygen permeability value changed little. Therefore, the barrier property of modified hemicellulose film will remain at a high level in the long term use.

The performance of modified hemicellulose film is basically acceptable for certain packaging purposes where mechanical properties is not emphasized.

## 5. Outlook

The origin, extraction and modification of hemicellulose all affect the physicochemical properties of hemicellulose films. The diversity of hemicellulose together with its available functionalities provides a good foundation for the development of hemicellulose film materials.

Main drawbacks of hemicellulose film with regard to its use as food packaging are poor mechanical properties and water sensitivity. Accordingly researchers have developed various methods combining physical and chemical modification to obtain hemicellulose composite films with good mechanical and barrier properties.

As future research direction, physical modification of hemicellulose film could focus on the compatibility of plasticizer and reinforcer with hemicellulose matrix to further improve the mechanical properties of the film. In terms of chemical modification, the main concern is to avoid degradation of hemicellulose caused by by-products. At the same time, in the study of bio-based materials, the combination with biodegradable materials such as poly lactic acid (PLA) should be considered to improve the biodegradation performance of composite materials, so as to truly make the production and abandonment process of materials environmentally friendly, and the life cycle of composite materials a green one.

Polysaccharide-based packaging film has been preliminarily applied in the field of food packaging. The application of hemicellulose film is still in the research stage. Its excellent barrier property has made hemicellulose film a potential alternative to plastic film where mechanical property is not particularly required, e.g., plastic wrap. Hemicellulose film can also be combined with other polymers, e.g., PVA to give full play to its barrier properties, and to reduce the use of petroleum-based materials.

## Figures and Tables

**Table 1:** The yield of hemicellulose using different extraction methods under different conditions.

Extraction Method	Reaction Condition	Yield (%)
Alkali or alkaline peroxide solution	2% NaOH, 90 °C	49.2
	2% NaOH, 60 °C	43.9
	2% H_2_O_2_, pH11.5, 90 °C	25.3
	4% H_2_O_2_, pH11.5, 90 °C	51.7
Two-step alkali extraction-delignification	2% H_2_O_2_, pH11.5, 60 °C	83.2
	4% H_2_O_2_, pH11.5, 60 °C	72.7
	0.75% NaClO, pH11.5, RT	70.9
	1.5% NaClO, pH11.5, RT	77.0

**Table 2:** Influence of different biomass on mechanical properties of thin films.

Biomass Source	Tensile Strength (MPa)	Elongation at Break (%)	Ref.
Bagasse	About 0.31~1.72	—	[26]
Cotton waste	1.3	—	[27]
Oil palm leaf	10	—	[28]
Barley bran	50	—	[29]
Norway spruce	55	2.7	[30]
Rape straw	About 101~218	About 3~85	[31]

**Table 3:** Effects of MMT, PVA, NCH, and CS as reinforcing agents on mechanical properties of the film.

Reinforcing Agent	Proportion of the Film	Tensile Strength (MPa)	Elongation at Break (%)
MMT	*V*(QH):*V*(MMT) = 1:1	19.8	0.5
MMT/PVA	*V*(QH):*V*(MMT):*V*(PVA) = 1:1:0.3	55.7	3.9
MMT/NCH	*V*(QH):*V*(MMT):*V*(NCH) = 1:1:0.3	28.6	2.3
MMT/PVA	*V*(QH):*V*(MMT):*V*(PVA) = 1:1:0.5	46.3	4.0
MMT/NCH	*V*(QH):*V*(MMT):*V*(NCH) = 1:1:0.5	42.1	3.8
MMT/CS	*V*(QH):*V*(MMT):*V*(CS) = 1:1:0.05	52.7	2.6
MMT/CS	*V*(QH):*V*(MMT):*V*(CS) = 1:1:0.08	57.8	2.8

**Table 4:** Effects of CNF on mechanical properties of the film.

CNF Mass Fraction (%)	Tensile Strength (MPa)	Elongation at Break (%)
0	11.9	3.4
5	15.5	2.9
10	20.2	2.6
15	28.9	1.8
20	39.5	1.4

**Table 5:** Effects of CNC on mechanical properties of the film.

CNC Mass Fraction (%)	Tensile Strength (MPa)	Elongation at Break (%)
0	9.87	3.25
1.2	12.29	3.35
6.8	15.47	3.13

**Table 6:** Comparison of mechanical properties and barrier properties between hemicellulose film and traditional film materials.

Film	Tensile Strength (MPa)	Elongation at Break (%)	Oxygen Permeability (cm^3^·μm/m^2^·d·kPa)	Moisture Barrier Property	Ref.
PET	45	335	58.34	WVTR: 1.49	[67]
PVDC	47.12	42.4	2.88	WVTR: 2.59	[68]
EVOH	40	230	2.77	WVTR: 0.60	[69,70,71,72]
Hemicellulose nanocomposite film	19.8–55.7	0.5–4.0	0.30–0.66	—	[36]
CA crosslinked hemicellulose film	2.0–10.0	5.7–44.4	1.8–5.4	—	[65]
PCL grafted hemicellulose film	3.9–15.4	11.5-40	—	—	[57]
LHs	9.78–33.94	2.97–22.41	1.21–4.24	WVP: 1.59–2.23	[48]
Borate crosslinking GM/GO film	78.95–135.54	8.82–9.62	0.11–0.25	WVP: 0.38–0.58	[66]

WVTR: Water vapour transmission rate (g/m^2^·d); WVP: water vapor permeability (10^−10^·g/m·s·Pa).
